# Durable antimicrobial activity of fabrics functionalized with zeolite ion-exchanged nanomaterials against *Staphylococcus aureus* and *Escherichia coli*

**DOI:** 10.3762/bjnano.17.18

**Published:** 2026-02-06

**Authors:** Perla Sánchez-López, Kendra Ramirez Acosta, Sergio Fuentes Moyado, Ruben Dario Cadena-Nava, Elena Smolentseva

**Affiliations:** 1 Universidad Nacional Autónoma de México, Centro de Nanociencias y Nanotecnología, Km. 107 Carretera Tijuana a Ensenada, C.P. 22860, Ensenada, Baja California, México; 2 Centro de Investigación Científica y de Educación Superior de Ensenada (CICESE), Carretera Ensenada-Tijuana, No. 3918, Zona Playitas, Ensenada 22860, México

**Keywords:** antimicrobial activity, *Escherichia coli*, fabrics functionalization, ion exchange, nanomaterial, *Staphylococcus aureus*, zeolite

## Abstract

Nanoparticle-based functionalization has emerged as an effective strategy to enhance the antimicrobial properties of textiles. In this study, silver (Ag^+^), copper (Cu^2+^), and zinc (Zn^2+^) cations are ion-exchanged with Y-type zeolite (CBV-600) and subsequently applied to cotton fabrics using the pad–dry–cure method, with an acrylic resin serving as binder. The resulting functionalized fabrics, containing metal cation concentrations of 1.0–1.5 atom % are evaluated regarding their antimicrobial activity against *Staphylococcus aureus* (Gram-positive) and *Escherichia coli* (Gram-negative), as well as regarding their physicochemical and mechanical properties. Scanning electron microscopy confirms the uniform distribution and successful incorporation of nanomaterials onto the fabric surfaces. Antimicrobial tests reveal significant inhibition of bacterial growth, with silver-based materials demonstrating superior efficacy. Importantly, the antimicrobial effect persists after five washing cycles, demonstrating the durability of the functionalization. This method demonstrates a simple and industry-compatible approach for producing durable antimicrobial cotton fabrics.

## Introduction

The development of nanotechnology has expanded into different areas of science, including physics, chemistry, biology, and medicine, over the past few decades [1–2]. Recently, nanoparticles (NPs), nanomaterials, and nanocomposites have been applied in various fields, including medicine and biotechnology, to reduce the recurrence of infections [3–4]. In this context, nanotechnology and nanomaterials offer a new alternative to combat pathogens such as viruses and bacteria. Metal NPs have intrinsic manipulatable properties that make them useful in a wide variety of research fields, including biomedicine. Different types of NPs are currently explored for various biomedical applications, including disease prevention, diagnosis, and the improvement of antiviral drug delivery systems [5–6]. In some cases, the antimicrobial properties of NPs lead to creation of new “nano-antimicrobial” materials [7]. The mechanism by which nanoparticles act against viruses involves the interaction with the surface of the NPs, leading to adhesion and inactivation, thereby preventing the virion from entering the host cell. NPs release ions or transfer them to microorganisms, inducing oxidative stress. Given these possible mechanisms, viral microorganisms are unable to develop mutations for adaptation and are destroyed [8–9].

Recently, several types of NPs, including silver, copper, and zinc, have demonstrated great potential in antimicrobial applications due to their properties such as high specific surface area, safety for human use, multiple synthesis methods, and relatively low cost [4,10–11]. One of the most extensively studied nanomaterials in terms of antimicrobial properties is colloidal silver as its antimicrobial action affects various parts of microorganisms. In addition to silver, copper has attracted significant attention for its antimicrobial properties. It was officially recognized in 2008 by the United States Environmental Protection Agency (EPA) as the first metallic antimicrobial agent, highlighting its potential for broad-spectrum antibacterial applications [12]. Since then, copper has been extensively studied for its ability to inactivate a wide range of microorganisms. Today, both silver and copper are widely used in various medical and healthcare applications due to their effective antimicrobial activity.

For example, copper was found to be 99.9% efficient in inactivating microorganisms within the first two hours of contact [13]. The antimicrobial activity of copper alloys (61–95% Cu) against *E. coli* O157 was tested at different temperatures (4 and 22 °C) [14]. The highest antibacterial effect was observed at 22 °C, but only alloys containing 95% of Cu completely killed *E. coli*. Chitosan–copper nanoparticles exhibited high antibacterial activity against various bacterial strains, including methicillin-resistant *Staphylococcus aureus* (*S. aureus*), *Bacillus subtilis*, *Pseudomonas aeruginosa*, and *Salmonella choleraesuis* [15]. Some studies have shown that the size of Cu NPs plays a crucial role in their antimicrobial activity. For example, CuO NPs with a size of 4.8 nm demonstrated better antibacterial activity than larger particles (7.8 nm) [16]. The small Cu NPs have a greater capacity to penetrate the cell membrane [17–18].

Zinc oxide NPs are well known for their photocatalytic properties. Also, recent studies have demonstrated that ZnO possesses unique antibacterial, antimicrobial, and antifungal properties, making it effective against both Gram-positive and Gram-negative bacteria [19–20].

Recently, the application of nanotechnology has been extended to textiles. Fabrics functionalized with nanoparticles, also known as “smart textiles”, possess a range of properties, including antistatic, UV-blocking, hydrophobic, electronic, thermoregulation, and antimicrobial effects [21–23]. One of the simplest way to incorporate nanomaterials into fabrics is by mimicking designs found in nature. It is well known that the leaves of some plants are water- or dust-repellent, and these repellent properties can be transferred to textiles by modifying the components involved in these mechanisms [24]. Among the physicochemical methods used for fabric functionalization with nanomaterials are coatings, electrospinning, assembling different components, fiber material composites, nanoscale fibers, and immersion in NPs solutions with a binder.

Nanoparticles such as Ag, Au, TiO_2_, ZnO, Se, SiO_2_, CuO, and Pt are widely used for textile functionalization [25]. Common methods for incorporating Ag and Cu NPs into cotton textiles involve treating the fibers at the end of the manufacturing process. Those methods require the use of previously prepared NPs, which are then bonded to the textile through chemical bonding or electrostatic interaction. The pad–dry–cure method is an effective alternative for applying nanoparticles to the surface of fabrics. In this process, crosslinking reactant, catalyst, softener, and other components are dried onto the fabric before the crosslinking reaction takes place during the curing stage [26–28]. Lateef et al. applied the pad–dry–cure method to functionalize commercial cotton and silk with Ag NPs using a self-cross-linking binder. The functionalized textiles were tested against *S. aureus*, *Escherichia coli* (*E. coli*), *Klebsiella pneumoniae*, *Klebsiella oxytoca*, *Proteus mirabilis*, and *Aspergillus niger*. It has been shown that at concentrations of 100 and 150 μg·mL^−1^, Ag NP-functionalized cotton and silk effectively inhibited the growth of the test isolates up to the fifth wash cycle [29]. Ag NPs deposited on the cotton fabrics via the pad–dry–cure process were tested for bactericidal activity against the Gram-positive *S. aureus* ATCC 25923 [30]. The results show that cotton fabric with concentrations of 10 and 20 ppm of Ag NPs exhibited strong bactericidal properties, reducing bacterial colonies by over 98%. Biogenic Ag NPs, obtained through fungal biosynthesis using extracellular filtrate of the epiphytic fungus *Bionectria ochroleuca* were incorporated into cotton and polyester fabrics using the pad–dry–cure method [28]. The silver-modified fabrics display antimicrobial activity against *S. aureus*, *E. coli*, *Candida albicans*, *Candida glabrata*, and *Candida parapsilosis*.

Copper oxide nanoparticles were deposited on the cotton fabric in two steps: first, through microencapsulation using an ionic gelation method and exhaustion, followed by the pad–dry–cure method in the second step. The antibacterial properties of the coated fabric were then evaluated [31].

Zinc peroxide (ZnO_2_) NPs synthesized via the sol–gel method were used to functionalize cotton textile fabrics through the pad–dry–cure method [32]. As shown in [33], ZnO NPs prepared through the sol–gel method were subsequently applied to the cotton fabric using the pad–dry–cure technique with dimethyloldihydroxyethyleneurea as a cross-linking agent. The results demonstrate good antibacterial activity against *S. aureus* and *E. coli* bacteria. The synthesis conditions, including concentration (1% and 2%), temperature (25 and 80 °C), and order of the ZnO NP application during the pad–dry–cure method were studied by Eskani and coworkers. Antibacterial activity of the treated fabrics was evaluated against *S. aureus* and *E. coli* [34].

Recently, the versatility of zeolite carriers for stabilizing silver–copper exchange zeolite microparticles demonstrating potential as durable antimicrobial agents for textile applications was highlighted [35].

Building on this growing body of research, our study focuses on the use of Ag, Cu, and Zn-exchanged Y-type zeolites, which offer high ion-exchange capacity and a well-defined framework structure that facilitate efficient antimicrobial ion delivery. By applying those nanomaterials to commercial cotton fabrics via the pad–dry–cure method, we aim to further explore the feasibility and effectiveness of zeolite-based antimicrobial treatment under conditions relevant to large-scale textile finishing. In the present work, the functionalization of textiles using the pad–dry–cure method with silver, copper, and zinc ions loaded on Y-zeolite (CBV-600) is demonstrated. The antimicrobial properties of impregnated textile samples were studied against *S. aureus* and *E. coli*, and their physicochemical and mechanical properties are discussed. The obtained results suggest that the synthesized materials can be applied as an effective alternative to inhibit and reduce the spread of different types of viruses and bacteria. Unlike our previous work, which focused on the antimicrobial performance of Ag- and Zn-loaded Y zeolite, the present study applies those nanomaterials, including Cu, to cotton fabrics via the pad–dry–cure method and evaluates the durability of their antimicrobial activity after fife washing cycles, representing a direct and practical extension of the prior findings.

## Results and Discussion

### Chemical composition

**Figure 1:**
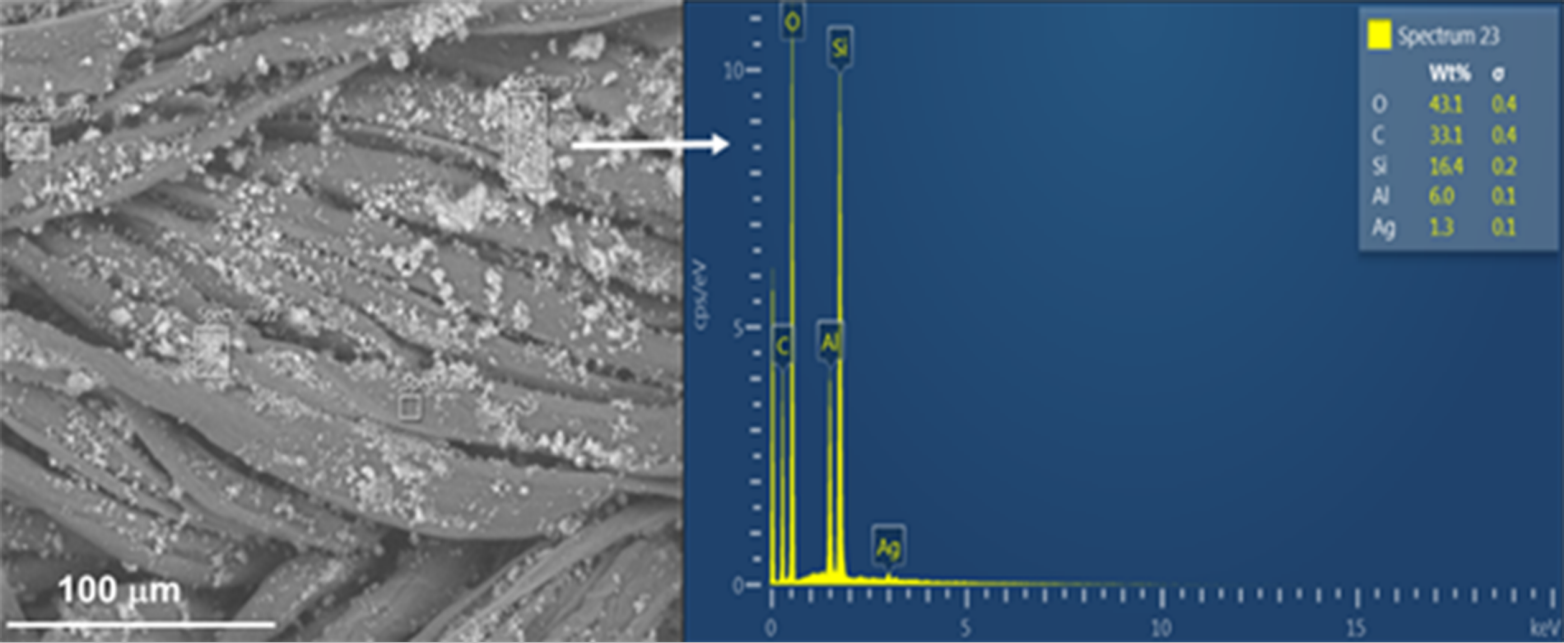
SEM micrographs of Ag/CBV-600-bramante (left) and its chemical analysis by EDS (right).

Prior to the fabric functionalization, the silver, copper, and zinc loadings in, respectively, Ag/CBV-600, Cu/CBV-600, and Zn/CBV-600 nanomaterials were analyzed by inductively coupled plasma optical emission spectroscopy (ICP-OES). The results confirmed silver, copper, and zinc contents of around 1.0–1.5 atom % [4]. Energy-dispersive X-ray spectroscopy (EDS) analysis performed on the functionalized fabrics in the present work confirmed the presence of silver (1.3 wt %), as well as copper and zinc (0.3 wt %) on the surface of textile (see Table 1 and Figure 1, right panel). The slight increase in silver content on the functionalized fabrics may be explained by the agglomeration of nanomaterials on the surface of fabrics and superficial nature of the EDS method. However, the content of copper and zinc in the functionalized textiles analyzed by EDS was nearly four times lower in comparison with Ag/CBV-600-bramante. This may be explained by non-homogeneous loading of nanomaterials on the textile, as also evidenced by SEM images. The Si/Al molar ratio for Y zeolite was 2.7, which coincided well with data provided by the supplier and measurements obtained through EDS and ICP-OES analysis. As discussed in our previous work [4], the ion exchange treatment did not induce any changes in the chemical composition of the zeolite structure, such as dealumination or disilation. No additional impurities in the samples were found.

**Table 1:** Contents of silver, copper, and zinc in the modified textile samples measured by EDS.

Sample	Metal loading, wt %					

	Si	Al	Ag	Zn	Cu	Si/Al

Ag/CBV-600-bramante	16.4	6.0	1.3	—	—	2.7
Cu/CBV-600-bramante	15.7	5.9	—	—	0.3	2.7
Zn/CBV-600-bramante	12.2	4.7	—	0.3	—	2.6

### SEM analysis

**Figure 2:**
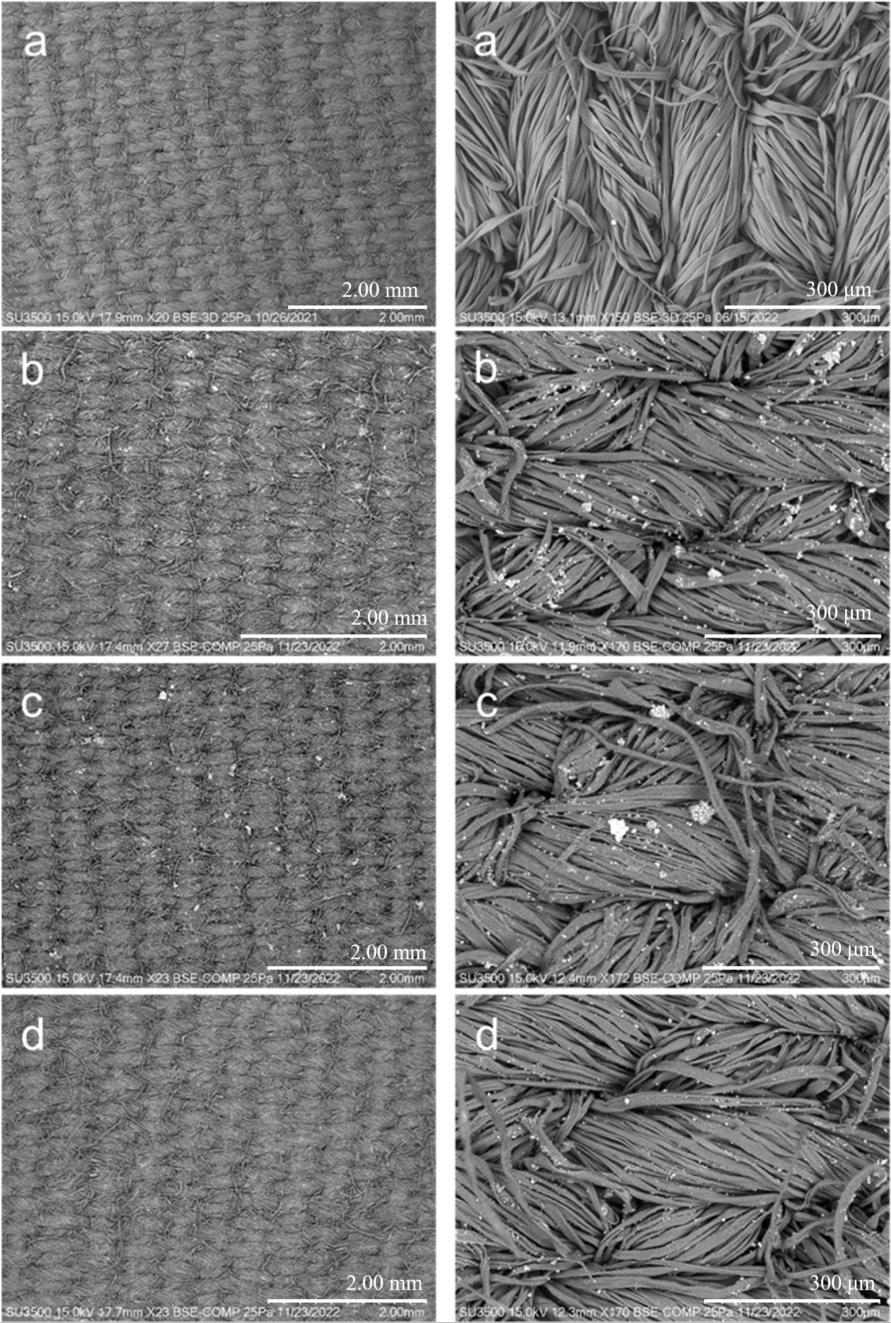
SEM micrographs of the samples. (Left column) Panoramic view and (right column) zoomed view. (a) Bramante fabric, (b) Ag/CBV-600-bramante, (c) Cu/CBV-600-bramante, and (d) Zn/CBV-600-bramante.

Figure 2 shows the micrographs of bramante fabric before and after its functionalization with 0.5 g of Ag/CBV-600, Cu/CBV-600, and Zn/CBV-600 nanomaterials using the pad–dry–cure method. As seen in Figure 2a, the original fabric did not show fiber degradation after washing prior to its functionalization. However, some foreign particles were observed, which may be attributed to contamination during handling (Figure 2a, right panel). These types of impurities are commonly present in fabrics, even after sterilization [28].

Once 0.5 g of nanomaterials (Ag/CBV-600 in Figure 2b, Cu/CBV-600 in Figure 2c, and Zn/CBV-600 in Figure 2d) were incorporated into the fabric, small, well-dispersed particles were observed on the textile surface. Panoramic micrographs of the functionalized fabrics showed varying degrees of nanomaterial aggregation: Ag/CBV-600 > Cu/CBV-600 > Zn/CBV-600. Large particles were observed for the bramante fabric with 0.5 g of Cu/CBV-600 (approximately 30 μm) (Figure 2c). However, better distribution of nanomaterials on the fabric surface was achieved for Ag/CBV-600 and Zn/CBV-600 (Figure 2b,d). Moreover, relatively small particles were found for Zn/CBV-600-bramante (Figure 2d) with uniform dispersion of nanomaterials on the fabric surface. Note, that the changes in the degree of aggregation may be attributed to the nature of the nanomaterial.

The functionalization of fabrics with acrylic resin as a binder allowed for the effective fixation of nanomaterials onto the fabrics. It is known that the high concentration of the binder could affect the antimicrobial activity of the fabrics [30]. Neither aggregation of the acrylic resin (10% *w*/*w*) nor degradation of the fibers after functionalization was observed for the prepared samples, demonstrating the optimal concentration (Figure 2). Therefore, it can be concluded that the proposed methodology, using acrylic resin at a 10% concentration, allowed for the effective incorporation of the nanomaterial, fixing it onto the fabrics without compromising the biocidal properties of functionalized textiles.

### X-ray diffraction

**Figure 3:**
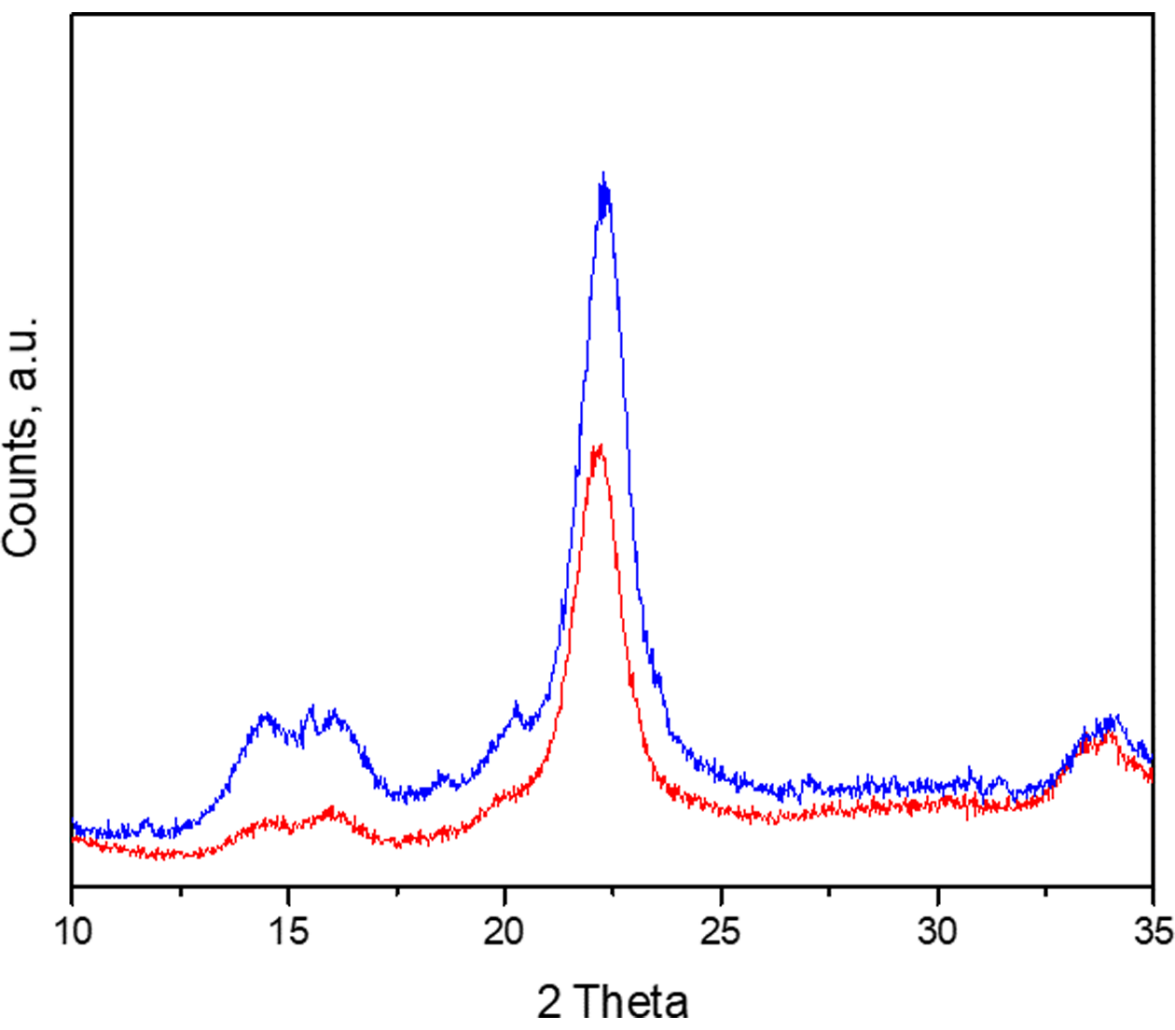
X-ray diffraction patterns of bramante fabric: nonmodified (red line) and modified with nanomaterial (blue line).

XRD patterns of bramante fabrics, composed of 50% cotton and 50% polyester fibers, are shown in Figure 3. The bramante fabrics exhibited a typical cotton cellulose pattern, with three characteristic peaks at 2θ ≈ 14.7°, 16.3°, and 22.4°, corresponding to the crystallographic planes (101), (101), and (002), respectively. Modification of the fabrics with nanomaterials led to an increase in the intensity of all peaks, while the position of the signals in XRD patterns remained unchanged (Figure 3). The later may be explained by the increase in crystallinity due to crosslinking with zeolite nanomaterials. A similar effect was observed in [36–37] for cotton fabrics cross-linked with dimethyloldihydroxyethyleneurea or treated with gallnut extract, respectively.

### Antibacterial activity of functionalized fabrics

**Figure 4:**
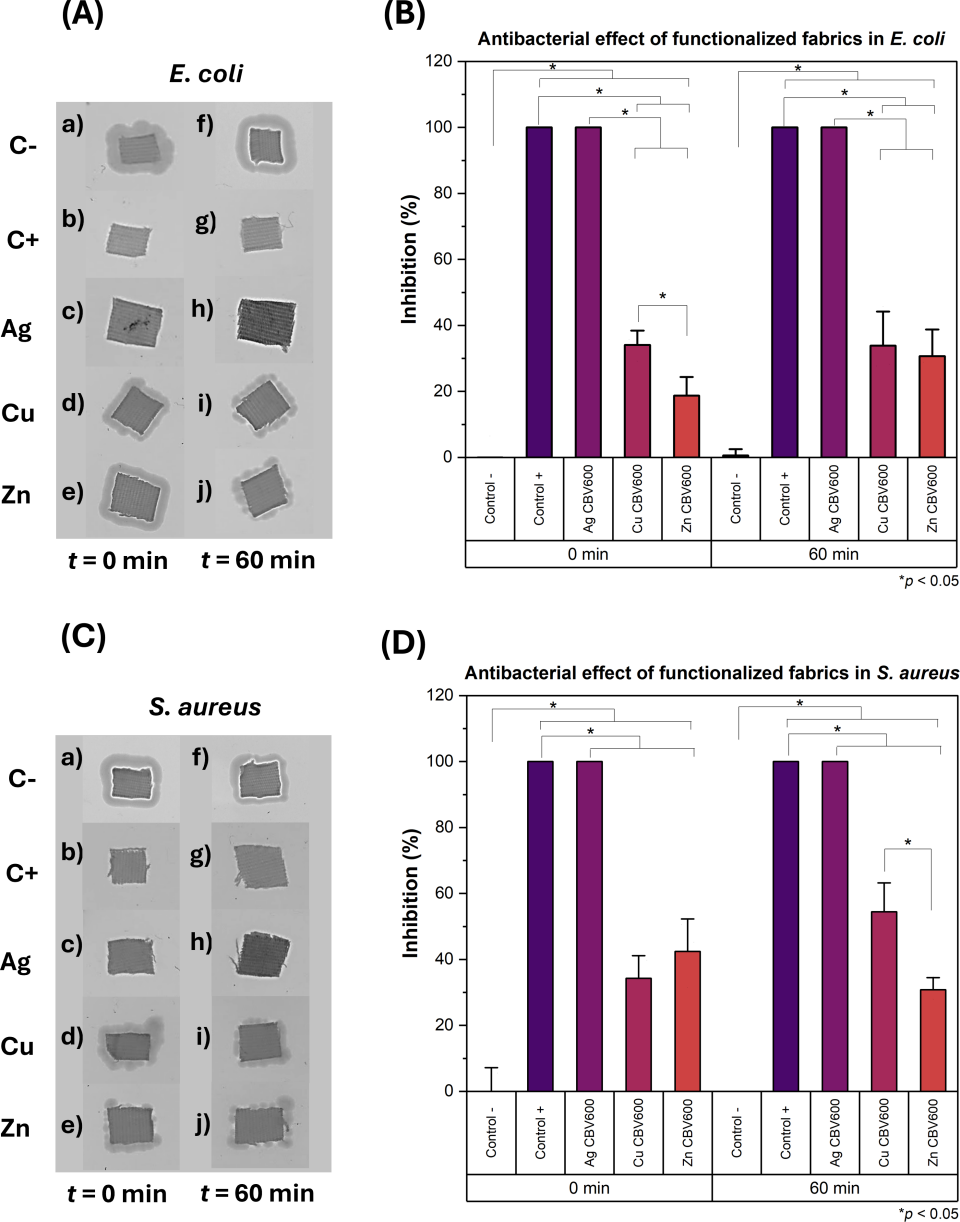
(A) *E. coli* growth on LB agar plates on fabrics exposed to 0 and 60 min of sunlight. (B) Inhibition of *E. coli* growth on fabrics after 0 and 60 min of exposure to sunlight. (C) *S. aureus* growth on LB agar plates on fabrics exposed to 0 and 60 min of sunlight. (D) Inhibition of *S. aureus* growth on fabrics after 0 and 60 min of exposure to sunlight. The asterisk (*) indicates *p* < 0.05.

During the sunlight exposure assay, groups (A) and (C) of fabrics, corresponding to *E. coli* and *S. aureus*, respectively, were exposed to visible light at an intensity of 96.9 klux and UV-A/B radiation at 6704 µW·cm^−2^ under a glass microscope slide (Figure 4 and Table 2).

**Table 2:** ANOVA results of antimicrobial effects of functionalized fabrics against *E. coli* and *S. aureus*.

Antimicrobial activity of functionalized fabrics against *E. coli*					

	DF	Sum of squares	Mean square	F value	P value
	
materials	4	50105.21	12526.30	437.18	<0.0001
sunlight exposure	1	45.61	45.61	1.59	0.22
model	5	50150.82	10030.16	350.06	<0.0001
error	24	687.67	28.65	—	—
corrected total	29	50838.49	—	—	—

Antimicrobial activity of functionalized fabrics against *S. aureus*					

	DF	Sum of squares	Mean square	F value	P value
	
materials	4	45116.36	11279.09	181.02	<0.0001
sunlight exposure	1	22.15	22.15	0.36	0.56
model	5	45138.51	9027.70	144.89	<0.0001
error	24	1495.37	62.31	—	—
corrected total	29	46633.89	—	—	—

As seen in Figure 4, Ag/CBV-600-bramante showed strong antibacterial activity against *E. coli* (Figure 4Ac and 4Ah) and *S. aureus* (Figure 4Cc and 4Ch), as indicated by the lack of bacterial growth around the fabric. This effect was observed even when the fabrics were not exposed to sunlight and can be attributed to the well-known, strong antimicrobial properties of silver [38–41]. The susceptibility of bacteria to antimicrobial materials may depend on several factors, such as dose, humidity, and strain specificity [42–43]. Silver has been shown to exert a biocidal effect similar to that of copper against several *E. coli* and *S. aureus* strains using lower doses [43]. Likewise, surfaces coated with silver have exhibited lower minimum inhibitory concentrations (MICs) against *E. coli* and *S. aureus* strains compared to surfaces doped with copper and zinc [44]. Considering this, it is worth noting that the weight percentage of Ag/CBV-600 in the fabrics is four times higher compared to Cu/CBV-600 and Zn/CBV-600 as shown in Table 1. This increase in the concentration of antimicrobial agent may also explain the difference in the antimicrobial activity observed compared to Cu/CBV-600 and Zn/CBV-600. If a greater weight content of Cu and Zn were to be obtained on functionalized fabrics, a similar antimicrobial effect to what is exhibited by Ag/CBV-600 might be achieved.

Although no direct comparison can be made between the effect of Ag/CBV-600-bramante with and without sunlight exposure, the impact of sunlight exposure on Ag/CBV-600-bramante was evident from the darkening of the fabrics that were exposed to sunlight (Figure 4Ah and Figure 4Ch). This suggests that the Ag^+^ ions and small Ag NPs localized on the surface may undergo oxidation due to sunlight exposure. Cu/CBV-600 and Zn/CBV-600 nanomaterials exhibited a weaker antibacterial effect compared to AgCBV-600, particularly against *E. coli* (Figure 4Ad,i,e,j), and there was no significant difference between fabrics that were exposed to sunlight and those that were not. However, the biofilm thickness around these fabrics was smaller compared to the biofilm observed on the bacteria control groups with untreated fabric (Figure 4Aa,f). In this case, the antibacterial activity remained similar regardless of whether the fabrics were exposed to sunlight, as seen in Figure 4A. For *S. aureus*, the antibacterial activity of fabrics functionalized with Cu/CBV-600 and Zn/CBV-600 remained weaker compared to Ag/CBV-600. Additionally, sunlight-exposed fabrics functionalized with Cu/CBV-600-bramante and Zn/CBV-600-bramante (Figure 4B) did not show any blackening. However, sample Cu/CBV-600-bramante could inhibit *S. aureus* growth by up to 50% (Figure 4D) when exposed to sunlight. This represents a 20% increase in biocidal activity compared to Cu/CBV-600-bramante fabrics that were not exposed to sunlight, suggesting that the photocatalytic activity of Cu enhances its antibacterial efficacy. For fabrics with Zn/CBV-600-bramante, a minor increase in the inhibition of bacterial growth was also observed for *E. coli* following sunlight exposure.

**Figure 5:**
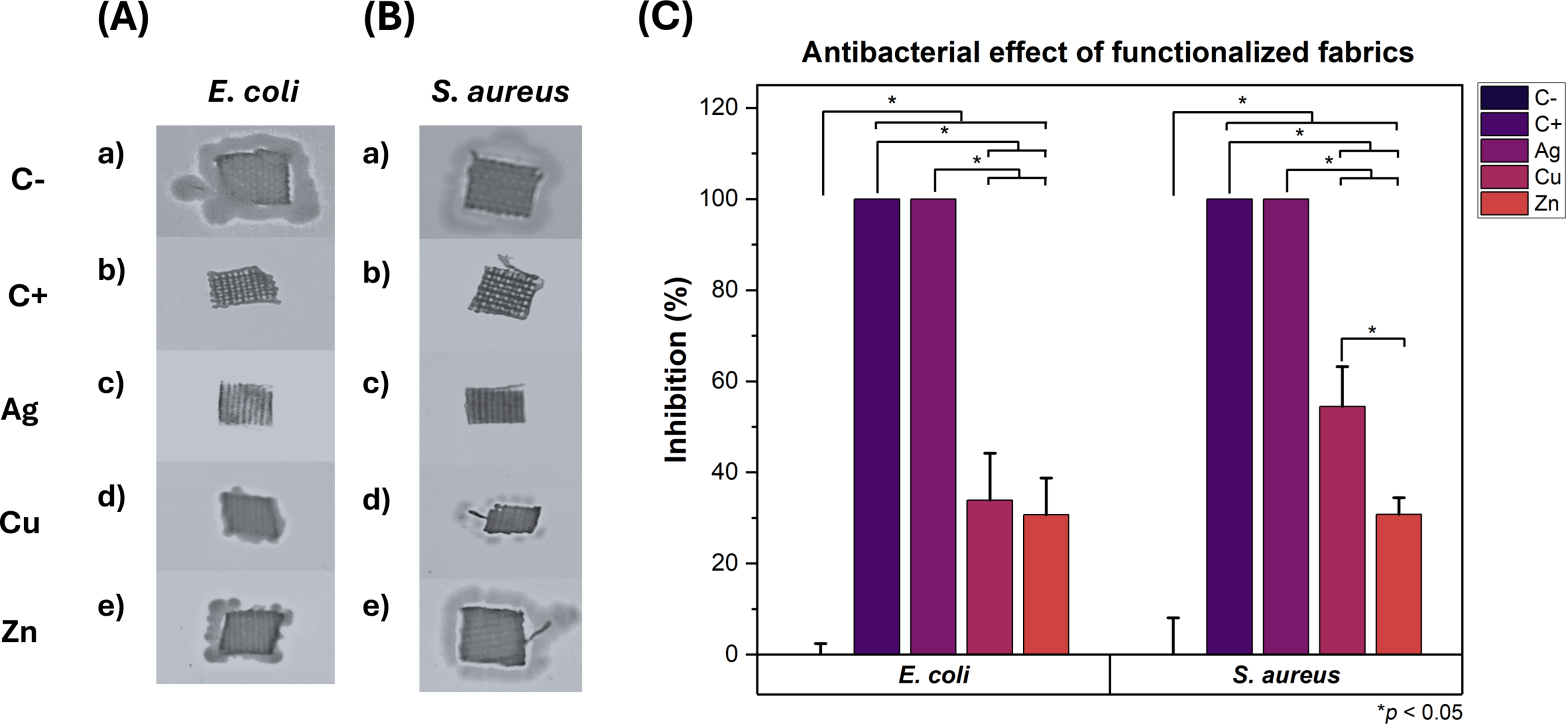
Antibacterial activity of fabrics after four wash cycles. Growth on LB agar plates of *E. coli* (A) and *S. aureus* (B) cultures after 20 h of incubation. Comparison of the inhibitory effect of fabrics impregnated with nanomaterials against *E. coli* and *S. aureus* (C). The asterisk (*) indicates *p* < 0.05.

Considering the effects observed on the fabrics exposed to sunlight, washed fabrics were exclusively tested under 60 min of sunlight exposure. A radiation level of 94.4 klux and UV-A/B radiation of 7.05 mW·cm^−2^ were registered. The antibacterial activity of fabrics after the fourth washing cycle is shown in Figure 5, along with the corresponding ANOVA results shown in Table 3. These fabrics were impregnated with *E. coli* or *S. aureus* and incubated on lysogeny broth (LB) agar plates for 20 h. The analysis of the growth radius around the fabrics shows that Ag/CBV-600-bramante fabrics inhibited bacterial growth by up to 95% (Figure 5A and Figure 5B). In contrast, Cu/CBV-600-bramante and Zn/CBV-600-bramante fabrics showed varying degrees of bacterial growth inhibition depending on the microorganism (Figure 5Ad,e and Figure 5Bd,e). Against *E. coli*, Cu/CBV-600-bramante fabrics inhibited bacterial growth by nearly 80%, while Zn/CBV-600-bramante fabrics achieved approximately 44% inhibition (Figure 5C). In contrast, the inhibitory effect of both Cu/CBV-600-bramante and Zn/CBV-600-bramante fabrics decreased against *S. aureus* after four cycles of washing. Bacterial growth was inhibited by 69% with Cu/CBV-600-bramante and by 16% with Zn/CBV-600-bramante. The stronger inhibition of Cu/CBV-600 compared to Zn/CBV-600 is consistent with results shown by Yao et al. [45], where Cu^2+^ zeolites showed higher inhibition of bacterial growth against *E. coli* and *S. aureus* compared to Zn^2+^ zeolites. The increased inhibition of *E. coli* and *S. aureus* using washed Cu/CBV-600-bramante may be attributed to the removal of excess material, which could lead to an increase in surface area and active sites [45]. Most importantly, these results demonstrate that the fabrics retain their antimicrobial activity even after four wash cycles. Such findings are significant for the potential applications of these fabrics in the manufacture of personal protective equipment.

**Table 3:** ANOVA results of antimicrobial effects of functionalized fabrics against *E. coli* and *S. aureus* after four wash cycles.

Antimicrobial activity of functionalized fabrics against *E. coli*					

	DF	Sum of squares	Mean square	F value	P value
	
materials	4	50105.21	12526.30	482.87	<0.0001
sunlight exposure	1	45.61	45.61	1.76	0.20
model	4	168.85	42.21	1.63	0.21
error	9	50319.67	5591.07	215.53	<0.0001
corrected total	20	518.82	25.94	—	—

Antimicrobial activity of functionalized fabrics against *S. aureus*					

	DF	Sum of squares	Mean square	F value	P value
	
materials	4	45116.36	11279.09	321.26	<0.0001
sunlight exposure	1	22.15	22.15	0.63	0.44
model	4	793.19	198.30	5.65	0.00329
error	9	45931.70	5103.52	145.36	<0.0001
corrected total	20	702.18	35.11	—	—

### SEM-EDS analysis of washed fabrics

**Figure 6:**
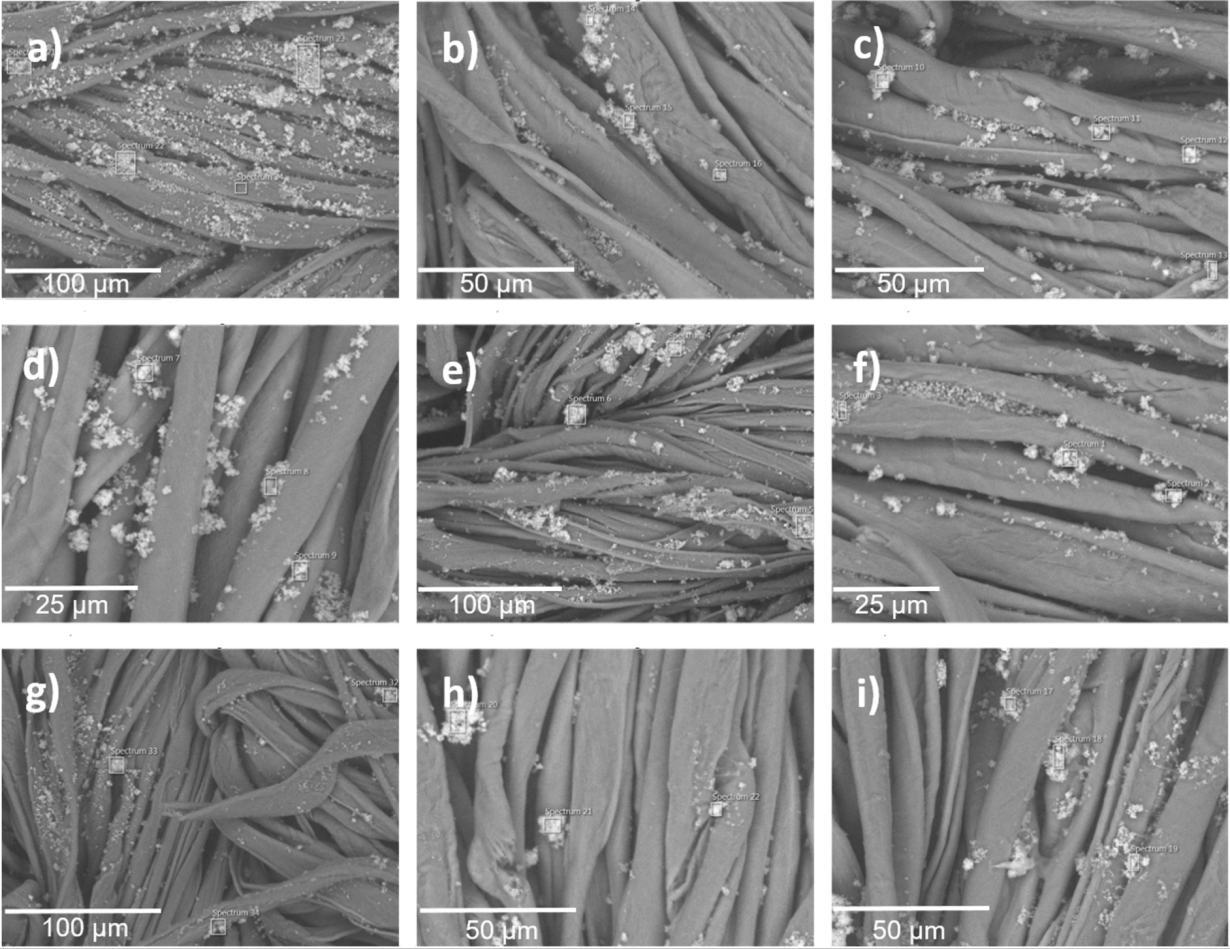
SEM micrographs of the samples after the first, third, and fifth washing cycles; (a–c) Ag/CBV600-bramante, (d–f) Cu/CBV600-bramante, and (g–i) Zn/CBV600-bramante.

**Figure 7:**
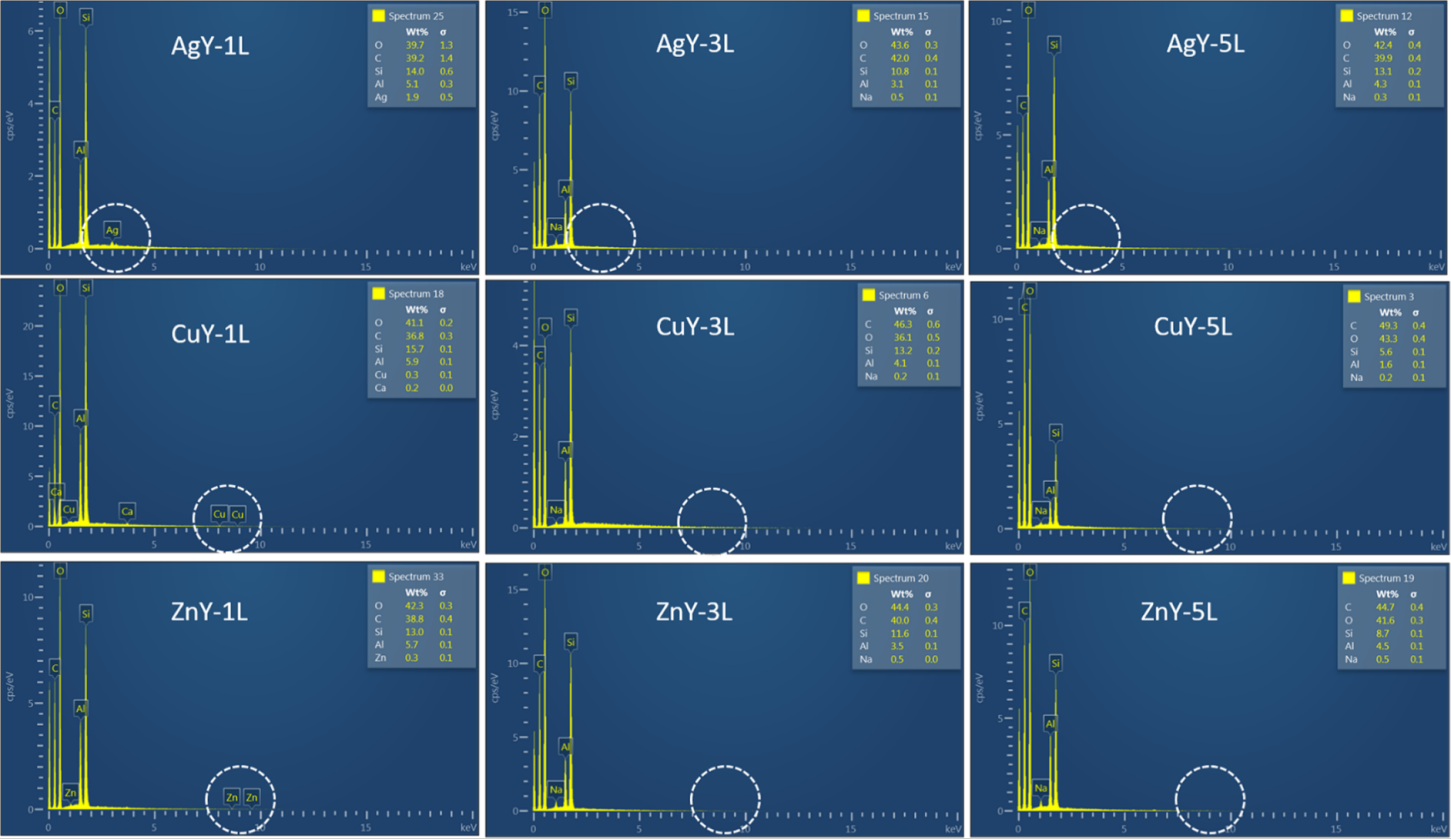
SEM-EDS analysis of bramante fabrics functionalized with Ag/CBV600, Cu/CBV600, and Zn/CBV600 after one, three, and five washing cycles.

To further evaluate the durability of the functionalized fabrics under practical conditions, the samples underwent repeated washing cycles, and their structural integrity and nanoparticles distribution were studied by SEM. Figure 6 shows the fabrics functionalized with the nanomaterials (Ag/CBV600-bramante, Cu/CBV600-bramante, and Zn/CBV600-bramante) after one, three, and five washing cycles. In all cases, the fabrics exhibited no fiber degradation after washing, and the presence of nanoparticles was still observed. However, the micrographs revealed different degrees of nanomaterial aggregation after each washing cycle. SEM-EDS analysis detected Ag, Cu, and Zn only after the first washing cycle (Figure 7). However, the Ag, Cu, and Zn concentrations after three and five washing cycles might be below the detection limit of EDS (i.e., the phenomenon is related to the relatively low amount of nanomaterials used for fabric functionalization rather than to the complete removal of the nanomaterials). According to the literature [30], partial nanoparticle loss during washing is commonly reported, with slight leaching occurring after five to ten washing cycles. This loss of nanomaterial may affect its antimicrobial properties; however, the useful life of the textile with antimicrobial properties is extended. The persistent antimicrobial activity observed after five washing cycles further supports the strong adhesion and stability of the metal-loaded zeolites on the fabric surface. Based on those experiments, only minimal metal leaching during washing is expected.

## Conclusion

This study successfully demonstrates the functionalization of cotton textiles using Ag^+^, Cu^2+^, and Zn^2+^ ions incorporated into Y-zeolite (CBV-600) via the pad–dry–cure method. The resulting nanostructured fabrics exhibited significant and durable antimicrobial activity against both *Staphylococcus aureus* (Gram-positive) and *Escherichia coli* (Gram-negative). Scanning electron microscopy confirmed the effective incorporation and uniform distribution of the nanomaterials on the fabric surface, facilitated by a 10% w/w acrylic resin binder. Among the tested materials, silver-based fabrics displayed the highest antibacterial efficacy, followed by copper and zinc, consistent with their known antimicrobial properties. Notably, the treated fabrics retained substantial biocidal activity even after multiple washing cycles, indicating the strong adhesion and stability of the zeolite-based nanomaterials. These findings highlight the potential of zeolite-supported metal ions as a scalable and effective approach for developing antimicrobial textiles with long-lasting performance. Such materials are promising candidates for use in healthcare, protective clothing, hygiene products, and other applications where microbial contamination poses a risk.

## Experimental

### Materials preparation

The pad–dry–cure method was used for fabric impregnation [30]. Prior to the experiment, the bramante fabrics containing 50% cotton and 50% polyester with a density of 150 threads (Parisina Company, Mexico) were washed with detergent (Alconox, Sigma Aldrich) and deionized water. Finally, the fabrics were dried at room temperature for one day. A 10% (*w*/*w*) acrylic resin (Acrylic binder 005, Royal Talens Company, Apeldoorn, Netherlands, chemical composition: 5-chloro-2-methyl-2*H*-isothiazol-3-one; 2-methyl-2*H*-isothiazol-3-one; 1,2-benzisothiazol-3(2*H*)-one) was used as a binding agent to immobilize the nanomaterials onto the fabrics. 100 mL of aqueous solution containing 0.5 g of M/CBV-600 (M = Ag, Cu, or Zn) suspension was prepared. Cut fabrics samples (5 cm^2^) were immersed once in the prepared solution for 15 min under constant stirring until full wetting. Then, each sample was compressed between two rolling pins using a rolling machine (Marcato, Padova, Italy). Afterwards, the functionalized fabrics underwent a drying process at 90 °C for 10 min; finally, the samples were cured at 120 °C for 3 min. Both stages were carried out in a mechanical convection oven (no. MMTUF110X2, Memmert GmbH, Germany). Prior to drying and curing, the fabric was characterized without nanomaterials and without acrylic resin, as shown in Figure 2a. The fabrics were functionalized using the ion-exchanged zeolite-based Ag/CBV-600, Cu/CBV-600, and Zn/CBV-600 nanomaterials prepared previously in [4]. A detailed physicochemical characterization of these materials, including metal content, morphology, and structural properties was reported in [4].

### Materials characterization

The morphology of the fabrics with and without NPs was analyzed by SEM. Images were acquired in a Hitachi SU3500 microscope operating at an accelerating voltage of 15 kV (Hitachi High-Tech Corp., Tokyo, Japan). The nanomaterial-containing fabric was placed on a metal grid with double-sided tape. For each sample, at least five representative images were captured. In order to analyze the grade of the washout of nanoparticles from the fabric, transmission electron microscopy (TEM) images were obtained using a JEOL JEM-2200FS (200 kV), and elemental analysis was performed using EDS.

The crystalline structure of the modified fabrics was determined by X-ray diffraction (XRD) in a Panalytical AERIS diffractometer using Cu Kα (λ = 1.54184 Å). The interval of XRD analysis was 2θ = 5–80°, with a step size of 0.01° and 1 s of measure time for each step.

The durability of the finish was assessed by washing the modified textile five times. The fabrics were washed using deionized water and the detergent Alconox. Mechanical action was applied using constant stirring at 5 rpm at 40 °C for 15 min per wash cycle, followed by drying at room temperature for 24 h between cycles. The antimicrobial properties of washed fabrics were evaluated after fourth washing cycle.

### Antibacterial activity of functionalized fabrics

Antimicrobial activity experiments against *E. coli* and *S. aureus* were conducted using a custom protocol based on the zone of inhibition method (ISO 20645) for hydrophobic fabrics. The experiments were performed using textile squares of approximately 0.5 × 0.5 cm^2^ of non-functionalized fabric (C− or negative control), fabric inoculated with 1 mg·mL^−1^ ampicillin (C+ or positive control), Ag/CBV600-bramante, Cu/CBV600-bramante, and Zn/CBV600-bramante. Bacteria cultures were prepared by growing them to a concentration of 10^8^ CFU·mL^−1^. Then, the fabrics were inoculated with bacteria by immersing each textile square for 5 s in 1 mL of culture (4 mL·cm^−2^ of fabric). Afterwards, the fabrics were deposited on top of a glass microscope slide (Fisher Brand) and covered with a second glass slide. This setup helped to prevent environmental contamination while the fabrics were exposed to sunlight. Two sample groups were evaluated to determine if the photocatalytic activity of the materials enhanced antimicrobial activity: group A was not exposed to sunlight (*t* = 0 min), and group B was exposed to 60 min of UV-A/B radiation of sunlight (*t* = 60 min), which was measured using an URCERI light meter containing a UV-A/B detector. Afterwards, all fabrics were deposited onto LB agar plates and incubated at 37 °C. Bacterial growth was determined by taking photographs of the plates after 20 h of incubation and measuring the biofilm growth on the sides of the fabric. The experiment was repeated using Ag/CBV-600, Cu/CBV-600, and Zn/CBV-600 bramante fabrics that were washed four times to determine if the antimicrobial properties were retained after several wash cycles. The images of bacterial growth were analyzed using ImageJ to determine the growth thickness around the fabrics. All assays were performed using three biological replicates per condition and 20 measurements of growth thickness were taken per sample. The average of these measurements was used to compare between biological replicates.

## Data Availability

Data generated and analyzed during this study is available from the corresponding author upon reasonable request.
